# Anorectal Gastrointestinal Stromal Tumor: Report of a Rare Case

**DOI:** 10.7759/cureus.38690

**Published:** 2023-05-07

**Authors:** Akash Patel, Akash Shah, Ishan Patel, Sunil Patel

**Affiliations:** 1 Medical School, Gujarat Cancer Society (GCS) Medical College and Hospital, Ahmedabad, IND; 2 Oncology, Apollo Hospitals, Ahmedabad, IND; 3 Medical School, Smt Nathiba Hargovandas Lakhmichand (NHL) Municipal Medical College, Ahmedabad, IND; 4 Pathology, Sterling Accuris Diagnostic, Ahmedabad, IND

**Keywords:** kit, gist, gastrointestinal, tumor, anorectal

## Abstract

Gastrointestinal stromal tumors (GIST) are mesenchymal spindle cell tumors of the gastrointestinal tract with the rarest occurrence in anal canal sites accounting for approximately only 2-8% of the anorectal GISTs. GISTs involve the expression of KIT (CD117) tyrosine kinase with the presence of mutations in KIT or platelet-derived growth factor alpha (PDGFRα) and are identified as an important target in therapy. The elderly population in the age of 70s appears to be at the highest risk with abdominal pain, GI bleeding, anemia, or weight loss as non-specific presenting symptoms. Here, we describe a case of a 56-year-old man who presented with vague dull pain in his left buttock diagnosed with GIST with a submucosal mass in the posterior wall of the anal canal and rectum and a tumor size of 45x42x37 mm. An immunohistological study of the biopsy sample reported positive for CD 117, CD 34, and DOG 1. The patient was prescribed neoadjuvant imatinib for 8 months with a good response and subsequently underwent transanal endoscopic microsurgical resection. Post-operatively, the patient was continued on adjuvant imatinib followed by regular restaging CT chest/abdomen/pelvis and surveillance flexible sigmoidoscopy every 6 months.

## Introduction

A gastrointestinal stromal tumor (GIST) is an infrequent mesenchymal neoplasm of the gastrointestinal tract originating from the interstitial cell of Cajal first described in 1983 [1]. Before two decades, it was considered leiomyoma, schwannoma, or leiomyosarcoma, but due to advances in molecular and immunohistochemistry, the diagnosis of GIST has become more precise and relatively easy. GIST has a very low incidence rate of around 1-2/100,000 people/year and a high rate of mortality with an overall survival rate of 11-30% [2]. GIST involves mutations of c-KIT proto-oncogene characterized by the expression of the KIT (CD-117) tyrosine kinase or platelet-derived growth factor receptor alpha (PDGFRα) [3]. Among different origins of GIST, anorectal GISTs are rarely detected as focal mural masses on CT imaging. Rectal GIST accounts for 0.6% of all rectal neoplasia and is a challenging diagnosis due to its rarity [4]. Patients with age greater than 50 are at higher risk of GIST with median diagnostic age of approximately 60 years [5]. Curative resection is considered the first-line treatment for localized GISTs, but it is difficult in rectal GIST due to anatomical barriers, including the deep, narrow pelvis and proximity to the sphincter muscle. Here we present a rare case of anorectal GIST in a 56-year-old male and its management outcome with a focus on pathological identification, site of origin, prognosis, and treatment.

## Case presentation

**Figure 1:**
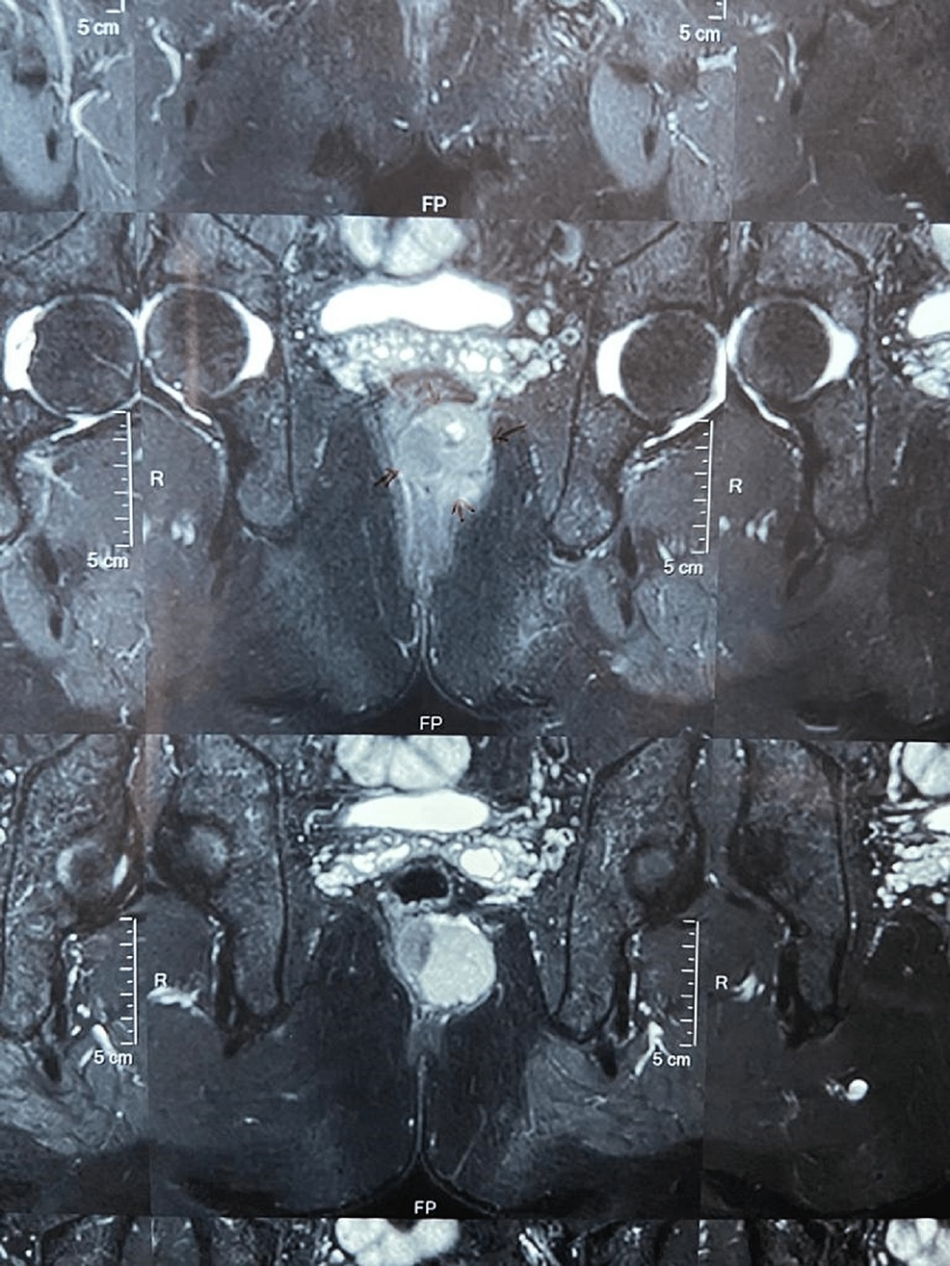
MRI indicating a well-circumscribed, solid, multilobulated lesion in the anal region on the left side with relation to the sphincter complex and projecting into the supralevator space.

A 56-year-old male presented with vague dull pain in the left buttock and the pain was increased by walking and forward bending. The patient did not have any dizziness, weakness, weight loss, or abdominal pain. There was no history of constipation or bleeding in stool. On clinical examination, no mass was observed in the pelvic and buttock region. Digital per rectal examination revealed a submucosal mass in the posterior wall of the anal canal and rectum present on the left side (2 to 7 o’clock position) and 1 cm away from the anal orifice. MRI of the pelvic region reported a single well-defined irregular-shaped lobulated mass situated at the anorectal junction left posteriorly which was found to be arising from the internal sphincter. The mass encroached upon the internal sphincter and compressed towards the posterior of the tumor resulting in severe pain and discomfort in passing the stool. The tumor size was 45x42x37 mm as per the MRI report (Figure 1).

PET scan showed no evidence of metastasis and no satellite lesion, and no lymphadenopathy was identified. 

**Figure 2:**
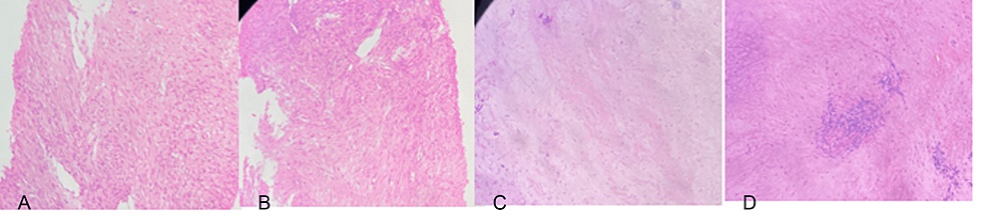
Histopathological finding of the mass using hematoxylin and eosin (H&E) stain with a magnification 100x shows neoplasm composed of loose fascicles and focal syncytial pattern of spindle-shaped cells with mild nuclear pleomorphism and arranged in sheets. (A) Pre-treatment histopathological finding of mass lesion using H&E stain 40x (B) Pre-treatment histopathological finding of neoplasm with loose fascicles and focal syncytial pattern of spindle-shaped cells using H&E stain 40x (C) Post-treatment histopathological finding of reduced neoplasm using H&E stain 40x (D) Post-treatment histopathological finding of reduced focal syncytial pattern of spindle-shaped cells using H&E stain 40x

A transrectal biopsy was performed and a true cut biopsy revealed the presence of GIST with low malignant potential with a mitotic activity of less than 5/50 HPF. Histopathological examination of tumor cells revealed that the tumor was 30x20x15 mm in size, hypocellular with large areas of hyalinization and sparsely placed spindle cells (Figure 2).

The viable tumor was approximately 5-10% and chemotherapy-related changes were approximately 90-95%. Immunochemistry analysis reported that the tumor cells were positive for CD117, CD34, and DOG1. In the genetic study, a mutation was observed positive in exon 11 while negative results for mutation were observed for exons 9, 13, 17, and PDGFRα.

Once the diagnosis was confirmed, the patient was started with neo-adjuvant targeted therapy imatinib mesylate 400 mg per day for downsizing the tumor. Within 10 days of imatinib treatment, the symptoms were relieved and the patient became asymptomatic. The patient was reassessed after one week with the CT scan, which showed a reduction in tumor size to 29x23x21 mm, and the treatment was continued for further 8 months with a continuous precise examination of the size of the tumor during neoadjuvant therapy with imatinib. Even after eight months there was no significant reduction in the size of the tumor. Hence, trans-anal minimally invasive surgery was planned for the patient. Preoperative sigmoidoscopy and manometry findings were found to be normal. The patient was operated on in the ninth month and the tumor was removed by the colonic submucosal tunneling and endoscopic resection (STER) method. No post-operative pain or leakage or defecation problems were observed after the resection of the tumor. The post-operative condition of the patient was significantly improved and the patient was discharged in stable hemodynamic condition. At the time of discharge, the patient was advised regular follow-ups with ultrasonography (USG) and MRI studies. Post-operative one-month sigmoidoscopy findings were normal with complete healing of the scar.

**Figure 3:**
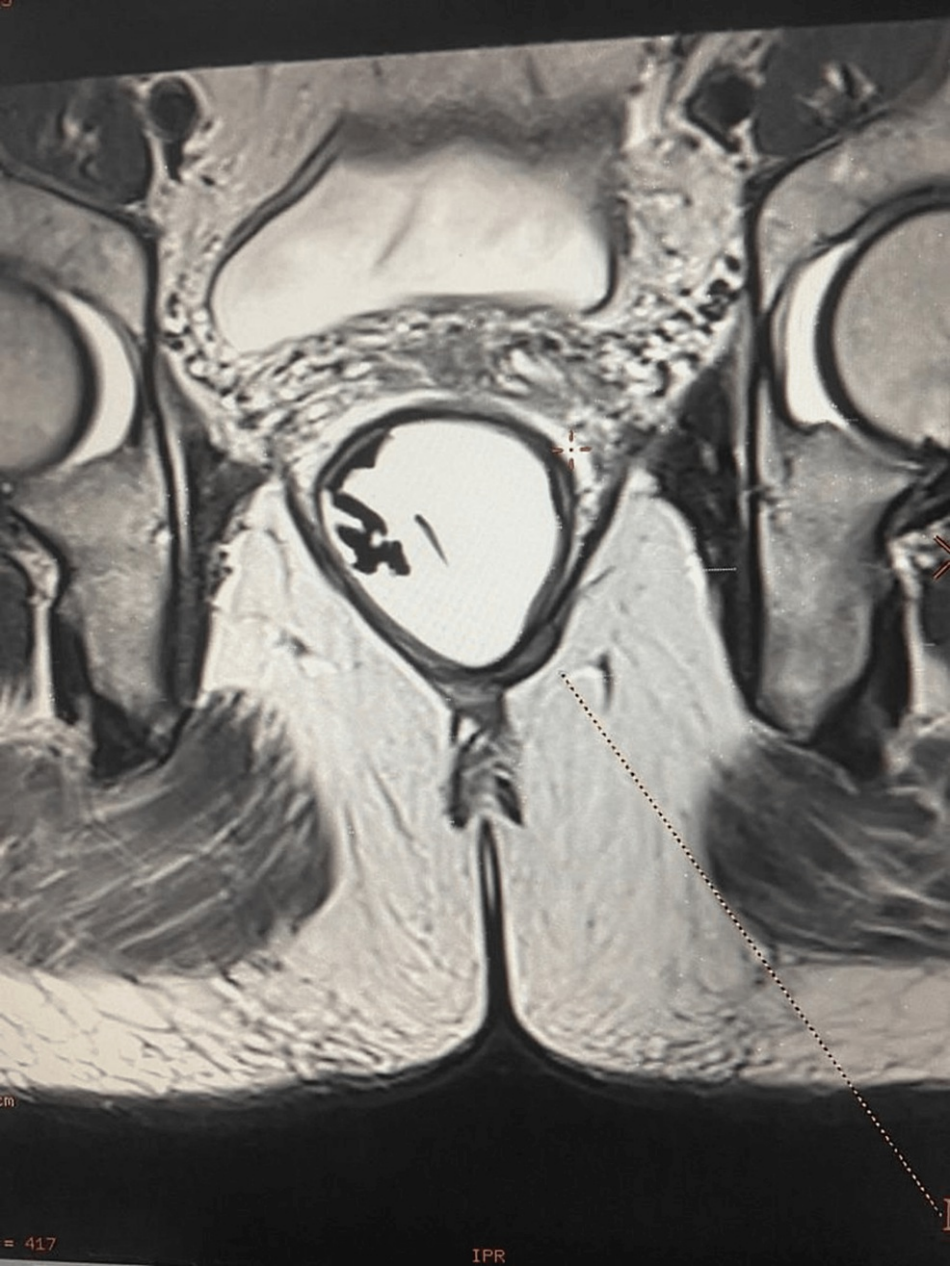
MRI finding of the residual lesion involving the left side of the anorectal junction adjacent to the intersphincteric space and the levator ani muscle.

Six months post-operatively, MRI showed a small ill-defined soft tissue lesion at the left side of the anorectal junction, adjacent to the intersphincteric space and abutting adjacent left levator ani muscle (Figure 3). The lesion extended from the 3 to 6 o'clock position. The lesion measured about 1.5x1.3 cm in the axial plane with a craniocaudal extension of 1.8 cm. The caudal end of the lesion was about 3 cm proximal to the anal verge, suggestive of fibrosis. No evidence of an extension of the lesion into the ischio-anal fossa or the supralevator space was found. PET scan showed non-fluorodeoxyglucose (FDG)-avid soft tissue lesion confirming fibrotic lesion.

## Discussion

GI stromal tumors are the rarest form of rectal cancers, occurring throughout the GI tract, from the lower esophagus to the anus, and account for 3% of anorectal mesenchymal tumors. The annual incidence of GIST is found to be approximately 1.1-1.4 per 100,000 population. Depending on the location the tumors in the anal canal, they can be either keratinizing or nonkeratinizing with similar biology and prognosis [6, 7].

GIST appears to arise from interstitial cells of Cajal or their stem cell precursors that regulate the autonomic functions of nerves and intestinal motility. The majority of GISTs are spindle cell tumors commonly characterized by the presence of spindle cells, with a small percentage of tumors possessing epithelioid histology. Mutations in the KIT proto-oncogene or PDGFRα are involved in GIST development. The expression of CD117, the antigen corresponding to the KIT protein, is considered to be a specific marker for the diagnosis of GIST. The majority of GIST cases were found to be CD117 positive followed by CD34 in an earlier reported case study [8]. Smooth muscle actin (SMA), desmin, and h-caldesmon (HCD) are smooth muscle markers predominantly expressed in GISTs of the small bowel and rarely expressed in the rectum [9]. Expression of smooth muscle markers is a differentiating and indicative factor for the diagnosis of leiomyomas and leiomyosarcomas which are the main tumors of GIST negative for KIT** **and CD34 and are often confused with GISTs. Mutation of exon 11 of KIT is often a ubiquitous feature of GISTs [10]. GIST tumors are often associated with low or relatively low mitotic rates and are graded based on mitotic activity. GIST tumors are often graded into two classes: low-grade tumors with a mitotic rate ≤5 per 50 HPF and high-grade tumors with a mitotic rate >5 per 50 HPF [11].

Contrast-enhanced CT scans and MRIs are the best modalities for the determination of the origin of the tumor, the surgical pelvic floor, the involvement of adjacent organs, and the tumor's distancefrom the anal verge. They also help assess the presence or absence of metastatic disease. Biopsy and immunohistochemical analysis of the biopsy sample for positivity of expressed CD117 and CD34 is considered the benchmark for precise and differential diagnosis of GIST tumors [12].

Combined treatment modality, including surgical resection and perioperative treatment with tyrosine kinase inhibitors-based systemic chemotherapy using imatinib mesylate and sunitinib malate, are gold standards for better management of GIST, facilitating anus-preserving surgery and associated with improved prognosis and survival.

## Conclusions

GIST is a rare and difficult-to-diagnose tumor of the gastrointestinal tract. It is associated with mutations of c-KIT, characterized by the expression of the KIT (CD-117) tyrosine kinase. Genetic analysis of this patient showed positive for mutation in exon 11 and negative for mutation in exon 9, 13, 17, and PDGFRα. Neoadjuvant chemotherapy with imatinib followed by surgical resection with colonic submucosal tunneling and endoscopic resection (STER) technique was the mainstay of the treatment, with a successful recovery of the patient.
